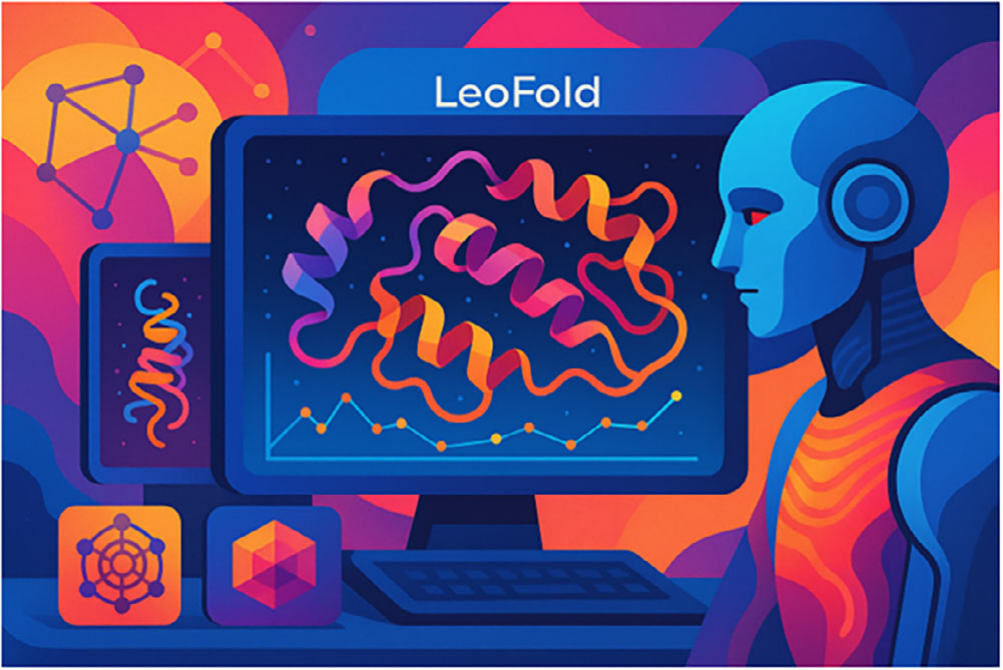# LeoFold: A 12‐Dimensional Conformational Rescue Platform for Late‐Stage Memory Recovery in Alzheimer's Disease – From Simulation to Clinical Signal

**DOI:** 10.1002/alz70861_108689

**Published:** 2025-12-23

**Authors:** Richard L Griffin

**Affiliations:** ^1^ LevelX, San Ramon, CA USA

## Abstract

**Background:**

In this presentation, we unveil the first clinical application of *LeoFold*, a high‐dimensional recursive attractor folding engine, capable of simulating and stabilizing the most damaged protein conformations observed in late‐stage AD patients.

**Method:**

Our research proposes a disruptive hypothesis: that protein misfolding is not only a symptom of AD, but also a reversible driver of cognitive collapse. This talk will provide scientific depth and public‐facing impact, framing AD not as a purely degenerative condition, but as a **foldable, recoverable system** with clinical and societal implications.

**Result:**

Think of a crumpled piece of paper—not just the shape it is now, but how it got that way. What stress folded it? What sequence of force unfolded it? LeoFold allows us to model folding not just in space, but in time. This is dynamic folding in four dimensions, shaped by disease.

LeoFold is a deterministic protein folding engine built upon a 12‐dimensional attractor field, simulating qubit‐stabilized protein folding paths in neurodegenerative systems. It is not a predictive tool alone, but a **therapeutic design engine**, identifying RMSD minima and entropy echo signatures required to force refolding in structurally damaged protein states.

**Conclusion:**

**Dynamic vs Static AI**: Unlike static models, LeoFold operates dynamically—tracking folding as a kinetic cascade, shaped by drugs like Fasudil, not only in structure but in folding history. This is not shape prediction; this is *shape evolution prediction*.

**Media‐Relevant Insight**:

• Alzheimer’s is no longer "irreversible."

• We have shown simulated reversal of memory‐linked protein collapse.

• LeoFold produces **target‐specific, genotype‐aware drug optimization** with path‐to‐clinic potential.

• In the age of generative AI, this is **generative biology** – creating therapeutic structure from cognitive collapse.

**Conclusion:**

This platform has broken the biological sound barrier of AD – not merely slowing decline, but **reversing it at the structural level**. LeoFold allows researchers to enter the folding landscape of Alzheimer’s proteins and **return with recovery maps**.

• **Scientific Quality**: Recursive qubit attractor modeling validated by thermodynamic convergence.

• **Relevance**: Targets AD where it was thought unchangeable – late stage.

• **Novelty**: First use of 12D echo‐stabilized RMSD tracking for therapeutic optimization.

• **Impact**: May redefine AD as a **recoverable synaptic topology disorder**, not a terminal neurodegeneration.